# Is Continuous Eruption Related to Periodontal Changes? A 16-Year Follow-up

**DOI:** 10.1177/0022034521999363

**Published:** 2021-03-03

**Authors:** C. Wiedemann, C. Pink, A. Daboul, S. Samietz, H. Völzke, E. Schulz-Kornas, K.F. Krey, B. Holtfreter, T. Kocher

**Affiliations:** 1 Department of Restorative Dentistry, Periodontology, Endodontology, Preventive Dentistry and Pedodontics, University Medicine Greifswald, Greifswald, Germany; 2 Department of Prosthetic Dentistry, Gerodontology and Biomaterials, University Medicine Greifswald, Greifswald, Germany; 3 Institute for Community Medicine, SHIP/Clinical-Epidemiological Research, University Medicine Greifswald, Greifswald, Germany; 4 German Centre for Cardiovascular Research (DZHK), Partner Site Greifswald, Greifswald, Germany; 5 Department of Cariology, Endodontology and Periodontology, University of Leipzig, Leipzig, Germany; 6 Department of Human Evolution, Max Planck Institute for Evolutionary Anthropology, Leipzig, Germany; 7 Department of Orthodontics and Dentofacial Orthopaedics, University Medicine Greifswald, Greifswald, Germany

**Keywords:** cohort study, tooth eruption, periodontitis, periodontal pocket, gingival recession, periodontal attachment loss

## Abstract

The aims of this study were to 1) determine if continuous eruption occurs in the maxillary teeth, 2) assess the magnitude of the continuous eruption, and 3) evaluate the effects of continuous eruption on the different periodontal parameters by using data from the population-based cohort of the Study of Health in Pomerania (SHIP). The jaw casts of 140 participants from the baseline (SHIP-0) and 16-y follow-up (SHIP-3) were digitized as 3-dimensional models. Robust reference points were set to match the tooth eruption stage at SHIP-0 and SHIP-3. Reference points were set on the occlusal surface of the contralateral premolar and molar teeth, the palatal fossa of an incisor, and the rugae of the hard palate. Reference points were combined to represent 3 virtual occlusal planes. Continuous eruption was measured as the mean height difference between the 3 planes and rugae fix points at SHIP-0 and SHIP-3. Probing depth, clinical attachment levels, gingiva above the cementoenamel junction (gingival height), and number of missing teeth were clinically assessed in the maxilla. Changes in periodontal variables were regressed onto changes in continuous eruption after adjustment for age, sex, number of filled teeth, and education or tooth wear. Continuous tooth eruption >1 mm over the 16 y was found in 4 of 140 adults and averaged to 0.33 mm, equaling 0.021 mm/y. In the total sample, an increase in continuous eruption was significantly associated with decreases in mean gingival height (*B* = −0.34; 95% CI, −0.65 to −0.03). In a subsample of participants without tooth loss, continuous eruption was negatively associated with PD. This study confirmed that continuous eruption is clearly detectable and may contribute to lower gingival heights in the maxilla.

## Introduction

Vertical eruption of human teeth predominantly occurs physiologically during the tooth formation period. The human dentition is considered a dynamic system being continuously exposed to masticatory and interdental forces. Here, we focus on continuous tooth eruption as one cause of interdental forces that can occur throughout life (Newman 1999). In the longer evolutionary perspective, continuous eruption might have evolved as a compensatory mechanism for heavy occlusal wear. Extensive tooth wear was ubiquitous in every past human population that consumed less refined and processed foods. However, in the last century, people of most modern societies have experienced a dramatic decrease in dental wear due to the consumption of softer and cleaned food items and differences in lifestyle (Benazzi et al. 2013).

It was suggested that occlusal tooth wear in ancient populations is highly linked to age (Miles 2001) but not necessarily to a reduction of the clinical crown (Levers and Darling 1983). In an ancient Romano-British population (Whittaker et al. 1982), the occlusal plane was maintained through continuous eruption, which compensated the wear and led to an increasing distance between the alveolar crest and the cementoenamel junction (CEJ). Additionally, radiographic studies on ancient human populations came to similar conclusions by using the inferior alveolar canal (IAC) as a reference and by showing that the IAC-CEJ distance and the distance between the IAC and the apices of the roots increased with age (Newman and Levers 1979; Levers and Darling 1983; Whittaker et al. 1990). However, the height distance between the occlusal surface and the alveolar bone crest was nearly unchanged across the age strata, implying a constant clinical crown height through life.

Evidence for continuous eruption can be found nowadays, although in modern human societies tooth wear due to dietary components is minimal. Replanted and ankylosed maxillary incisors submerge between their neighboring teeth (Kawanami et al. 1999), even after craniofacial growth has ceased. In a sample of young adults, infraposition of implant-borne restorations in relation to the upper front teeth continuously increased over 5 y (Thilander et al. 1999). Similarly, Bernard et al. (2004) observed a tooth eruption of up to 1.86 mm adjacent to implant-supported restorations after 4.2 y. A recent meta-analysis of implant-borne restorations based on 6 studies found a pooled prevalence of 20.8% for infrapositions >1 mm for implant restorations (Papageorgiou et al. 2018). Thus, continuous eruption does not only happen as a compensatory mechanism for tooth wear but has to be regarded as a physiologic feature, even without severe tooth wear. This was supported by measurements on maxillary casts that confirmed a steady increase in the palatal vault height (Thilander 2009).

If tooth eruption exists, there is also a continuous apical migration of the periodontal ligament, involving recession and attachment loss. Other groups disputed this hypothesis because the dentoalveolar height assessed on cephalometric images was smaller in younger adults than in middle and older ones (Tallgren and Solow 1991) and the gingival width increased over time (Mazeland 1980; Ainamo et al. 1981). These observations were interpreted to support the assumption that continuous eruption is an active process induced by vertical growth of the alveolar process itself and by the periodontal ligament shifting coronally with the elongated tooth. It is tenable now that different mechanisms can explain how continuous eruption might occur and to what extent it contributes to the loss of gingival attachment as reflected in periodontitis.

From the fifth decade onward, attachment loss is mostly driven by recession and less by pocketing (Holtfreter et al. 2009). Thus, gingival recession or an exposed root is commonly accepted as a characteristic feature of periodontitis and regarded as the result of a microbiologically driven inflammatory process (Lamster et al. 2016). As set out here, continuous eruption might be considered a contributing factor for gingival recession. On premodern skulls with exposed CEJ and root cementum, no typical signs of periodontal inflammation could be detected, such as a porous appearance of the alveolar housing or the loss of a crestal knife–edged contour (Clarke 1993). Thus, there may be another pathway leading to attachment loss and exposed roots besides periodontal inflammation. We assume that identifying continuous eruption as one factor contributing to recession and attachment loss could demand a reassessment of the high prevalence of stage I (incipient) periodontitis in seniors in modern populations.

So far, no study has concomitantly examined continuous eruption and periodontal variables with a reasonable sample size. Thus, using prospective cohort study data, we 1) examined if there is vertical continuous eruption of maxillary teeth in an adult modern human population over a 16-y period; 2) assessed the magnitude of such continuous eruption; and 3) measured effects of continuous eruption on changes in probing depth (PD), clinical attachment levels (CALs), gingival height (GH), and tooth loss by using data from the population-based prospective cohort of the Study of Health in Pomerania (SHIP).

## Materials and Methods

### Study Design

SHIP is a population-based study in the northeast of Germany (John et al. 2001). A 2-stage cluster-sampling procedure was adapted from the World Health Organization’s MONICA project (Monitoring Trends and Determinants in Cardiovascular Disease; Keil et al. 1988), gaining a representative sample of 7,006 Caucasian residents aged 20 to 79 y. Selection was carried out in proportion to a population of communities, stratified by age and sex. After the exclusion of deceased (*n* = 126) and migrated (*n* = 615) persons, the random sample included 6,265 participants. Of those, 4,308 aged 20 to 81 y were examined between 1997 and 2001 (baseline; SHIP-0), corresponding to a response rate of 68.8%. At the 16-y follow-up (SHIP-3), 1,718 participants were reexamined. All gave their written informed consent, and the study was approved by the local ethics committee (SHIP-0, issued July 31, 1995; SHIP-3, BB-122/13). The study was conducted and reported in accordance with the STROBE guidelines (Strengthening the Reporting of Observational Studies in Epidemiology). Detailed information on covariates is provided in the Appendix.

### Oral Examinations

Periodontal assessments included PD, CAL, and GH (Hensel et al. 2003). The periodontal examination was alternately carried out on the left or right side but always on the same side for the same participant. CAL and PD were assessed with a periodontal probe (PCP-11; Hu-Friedy) at the mesiobuccal, distobuccal, midbuccal, and midlingual/midpalatinal aspects on each selected tooth. CAL is represented by the distance from the CEJ to the pocket base. PD represents the distance from the gingival margin to the periodontal pocket base. GH represents the difference between PD and CAL. Measurements were mathematically rounded to whole millimeters. Only maxillary measurements were considered for calculations of mean PD, extent PD ≥4 mm, mean CAL, extent CAL ≥3 mm, and mean gingiva above the CEJ. Also, incidentally extracted teeth were excluded from these calculations.

A detailed description of the caries examination and derivation of the number of filled teeth is provided in the Appendix. All fully erupted teeth were counted, excluding third molars. In SHIP-0, tooth wear was assessed by the Ekfeldt index (Ekfeldt et al. 1990).

Dental examinations were conducted by calibrated and licensed dentists. Every 6 to 12 mo, calibration exercises were performed on persons not connected to the study. In SHIP-0, intrarater correlations for CAL were 0.82 to 0.91, and an interrater correlation of 0.84 was found. In SHIP-3, intrarater correlations for CAL were 0.92 to 0.97, while the interrater correlation was 0.98. For PD, intrarater correlations were 0.69 to 0.92, and the interrater correlation was 0.91.

### Assessment of Continuous Eruption

**Figure 1.**
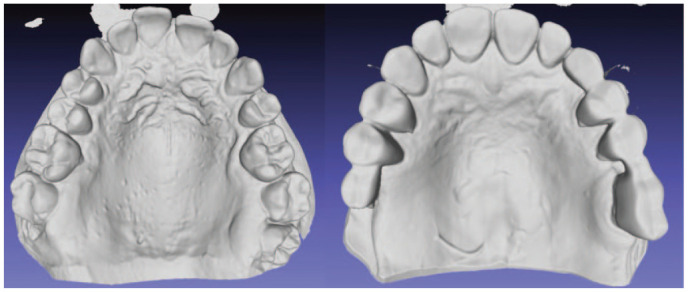
Occlusal view of usable (left) and unusable (right) models displaying the 3-dimensional surface of the triangulated networks from the MeshLab software.

**Figure 2.**
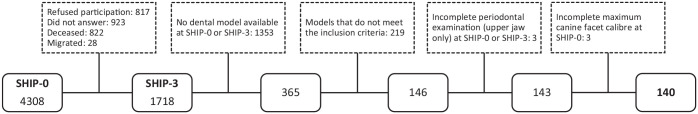
Flowchart describing the derivation of the study sample. SHIP, Study of Health in Pomerania; SHIP-0, baseline; SHIP-3, follow-up at 16 y.

At SHIP-0, 666 of 1,525 invited participants agreed, and alginate impressions (Palgaflex; Espe) were taken from both jaws to produce plaster casts. At SHIP-3, 365 participants accepted a repeated impression via StatusBlue (DMG Chemisch-Pharmazeutische Fabrik GmbH). In total, 365 pairs of plaster casts (type IV dental stone, high strength [expansion <0.10%]; Sherahard-Rock [Shera]) were available for analyzing the changes over this time. Casts were digitized with a 3-dimensional (3D) scanner (scan precision <10 µm; S600 [ARTI]) and the software Zirkonzahn.Scan (version 7212; Zirkonzahn GmbH). We restricted our analysis to 3D surface models of upper casts, because no immutable fixed reference points were detectable in the lower jaw. These 365 pairs of digitized models of the upper jaw taken at SHIP-0 and SHIP-3 were scrutinized for the following: 1) if the occlusal and palatal tooth area of the preselected teeth and their corresponding reference points were not altered and 2) if reference points on the occlusal and palatal area or rugae could be unequivocally determined on both digitized casts (Fig. 1). In total, 219 models were excluded. In addition, 3 participants had incomplete periodontal examinations, resulting in a final sample of 140 (Fig. 2).

**Figure 3.**
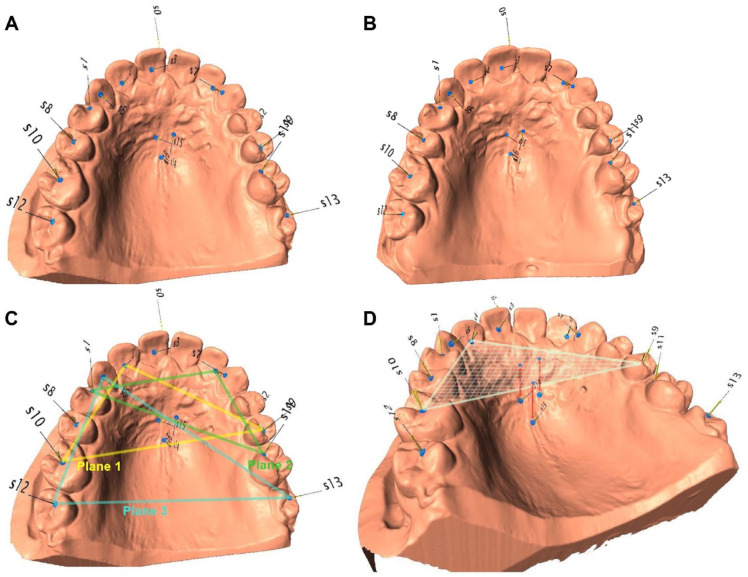
Visualization of the method. (**A**) Occlusal view of 3-dimensional surface model from SHIP-0 indicating the position of the reference points. (**B**) Corresponding model from SHIP-3 of the same participants: reference points were placed only on areas that did not change during follow-up. (**C**) Visualization of the 3 occlusal planes (Nos. 1 to 3). (**D**) Visualization of the vertical distances that are perpendicular between the occlusal plane (No. 1) and the palatal reference points. SHIP, Study of Health in Pomerania; SHIP-0, baseline; SHIP-3, follow-up at 16 y.

Since the palatal rugae are tightly fixed to the underlying periosteum, they were selected as reference points (Christou and Kiliaridis 2008; Chen et al. 2011). We used the medial ends of the most distal palatal rugae and 1 clearly recognizable fixed point on the distal area of the median palatine raphe as reference points (Fig. 3A, B). Additionally, 14 occlusal reference points on the teeth were defined (Appendix Table 1).

The occlusal (*n* = 14) and palatal (*n* = 3) reference points were simultaneously defined on both casts of each participant in the program Landmark (version 3.0.0.6; Institute for Data Analysis and Visualization) and had to be clearly ascertainable to ensure that SHIP-0 and SHIP-3 measurements were taken with the same reference points and occlusal planes. By doing so, we circumvented the impact of potential incident restorative treatment on the measurement of the vertical dimension during the 16-y interval (Fig. 3A, B). To construct the occlusal planes, 2 points on the occlusal surface of contralateral premolars or molars and 1 point in the fossa of a front tooth were manually selected per plane (Fig. 3C). To reduce the impact of single-tooth migration, 3 occlusal planes were defined, if possible, each with 3 teeth (Appendix Table 2).

To assess the height of the palatal vault, we measured the perpendicular distance from the occlusal planes to the palate reference points (Fig. 3D). This length was computed in Microsoft Excel 2016 via vector analysis and constituted the palatal vault depth. The vertical change (later termed *continuous eruption*) equaled the difference in the palatal vault depth between SHIP-0 and SHIP-3 cast models. For each occlusal plane, these changes of the distances to each palatal reference point were averaged, and the mean change of the 3 planes defined the level of continuous eruption on a participant level. To indicate tooth wear, the canine facet was gauged by a ruler tool in the Landmark software.

Repeated assessments of digitized 3D surface models of 30 participants (for SHIP-0 and SHIP-3 casts) were used to assess intrarater correlation coefficients for distances between the palatal reference points and the occlusal planes. Depending on the occlusal plane, reference point, and time point, intrarater correlation coefficients ranged from 0.58 to 0.85.

### Statistical Analyses

Means and standard deviations were computed for continuous variables and numbers (percentages) for categorical variables. To compare distributional differences between SHIP-0 and SHIP-3, paired *t* tests, McNemar’s tests, and Wilcoxon signed rank tests were applied.

Different regressions models were applied to evaluate the effects of 16-y changes in point-to-plane distances on 16-y changes in periodontal outcomes. Linear regression models with robust standard errors were used to model mean PD, extent PD ≥4 mm, mean CAL, extent CAL ≥3 mm, and mean gingiva above the CEJ. Linear regression coefficients (*B*) with 95% CIs and *P* values were reported. For modeling of any tooth loss (no/yes; 14 participants lost at least 1 tooth) within 16 y, logistic regression models were applied, and odds ratios with 95% CIs were reported. For the exposure variable, linearity assumptions were graphically assessed after locally weighted scatterplot smoothing. Additionally, models including linear terms or restricted cubic splines with 3 knots were compared by Bayesian information criterion and the best parameterization was applied accordingly.

To minimize bias and to avoid overadjustment, directed acyclic graphs (DAGs) were used (Akinkugbe et al. 2016). The platform DAGitty (Textor et al. 2011) was used to create various DAGs and to determine the most appropriate minimal sufficient adjustment set. First, we assumed that tooth wear was related only to continuous eruption but not to periodontal status (Appendix Fig. 1). The according adjustment sets included either {age, sex, number of filled teeth, education} or {age, sex, number of filled teeth, tooth wear}. Second, because occlusal forces might eventually have an effect on the periodontal status (Harrel et al. 2006), a relation between tooth wear and periodontitis was added to the DAG (Appendix Fig. 2). The according adjustment set again included {age, sex, number of filled teeth, tooth wear}. Thus, model 1 included age, sex, education, and number of filled teeth. For models 2 and 3, tooth wear was reflected by the Ekfeldt score (baseline levels; model 2) and the maximum canine facet caliber (baseline levels; model 3). Third, though not suggested by the DAG but often requested, models were additionally adjusted for periodontitis risk factors (model 4). Continuous confounders were modeled via restricted cubic splines with 3 knots.

Incident tooth loss might cause elongation or tipping of teeth and therefore bias point-to-plane measurements. Thus, we conducted sensitivity analyses excluding 27 participants with incident tooth loss.

Two-sided *P* values <0.05 were considered statistically significant. All analyses were performed with Stata/SE 16.1 (StataCorp 2019).

## Results

On average, participants in the selected subsample were 17 y younger and healthier than participants from the complete cohort with regard to all oral variables, and they had a more favorable risk profile (Table 1). This became particularly clear when we compared the dentate participants in the complete SHIP-0 cohort (*n* = 3,014) with those in the selected subsample (*n* = 140).

**Table 1.** Characteristics of the Complete Cohort, Study Population, and Subsample of Participants Without Incident Tooth Loss: Baseline (SHIP-0) and 16-y Follow-up (SHIP-3).

	Complete Cohort	Study Population			Participants without Incident Tooth Loss		
Variable	SHIP-0	SHIP-0	SHIP-3	*P* Value	SHIP-0	SHIP-3	*P* Value
**No. of participants** ^a^	4,254	140	140		113	113	
Follow-up time, y			16.0 ± 0.5			16.0 ± 0.5	
Age, y	49.8 ± 16.4	32.4 ± 8.2	48.5 ± 8.2	<0.001	32.6 ± 8.4	48.7 ± 8.3	
Male sex	2,091 (49.2)	68 (48.6)			53 (46.9)		
School education							
<10 y	1,706 (40.1)	7 (5.0)			5 (4.4)		
10 y	1,851 (43.5)	87 (62.1)			70 (62.0)		
>10 y	696 (16.4)	46 (32.9)			38 (33.6)		
Smoking status				0.39			0.55
Never smoker	1,527 (35.9)	60 (42.9)	56 (40.0)		51 (45.1)	48 (42.5)	
Ex-smoker	1,443 (33.9)	39 (27.9)	58 (41.4)		33 (29.2)	46 (40.7)	
Current smoker	1,284 (30.2)	41 (29.3)	26 (18.6)		29 (25.7)	19 (16.8)	
Abdominal obesity, yes	1,316 (30.9)	24 (17.1)	49 (35.0)	<0.001	18 (15.9)	38 (33.6)	<0.001
Diabetes mellitus, yes	467 (11.0)	3 (2.1)	10 (7.1)	0.04	3 (2.7)	9 (8.0)	0.07
No. of teeth	17.6 ± 9.6	26.7 ± 1.6	26.5 ± 1.7	<0.001	26.7 ± 1.5	26.7 ± 1.5	
**No. of participants** ^b^	3,014	140	140				
Dental checkup in last year, yes	2,718 (90.2)	123 (87.9)	125 (89.3)	0.84	101 (89.4)	103 (91.2)	0.81
Toothbrushig frequency ≥2/d, yes	2,530 (83.9)	116 (82.9)	120 (85.7)	0.45	98 (86.7)	98 (86.7)	1.00
Orthodontic treatment, yes	576 (19.1)	43 (30.7)			35 (31.0)		
Ekfeldt score	11.1 ± 8.5	9.0 ± 5.1			8.7 ± 4.8		
Maximum canine facet caliber, mm		3.4 ± 1.2	3.8 ± 1.3	<0.001	3.5 ± 1.2	3.8 ± 1.2	<0.001
Point-to-plane distance, mm		12.5 ± 2.3	12.8 ± 2.3	<0.001	12.5 ± 2.4	12.8 ± 2.4	<0.001
Restricted to maxilla							
No. of teeth	11.1 ± 3.0	13.5 ± 0.7	13.4 ± 0.8	<0.001	13.5 ± 0.7	13.5 ± 0.7	
PD, mm	2.50 ± 0.76	2.03 ± 0.39	2.31 ± 0.41	<0.001	2.02 ± 0.37	2.32 ± 0.43	<0.001
Extent PD ≥4 mm, %	12.3 ± 18.0	3.1 ± 6.0	6.6 ± 10.0	<0.001	3.0 ± 5.5	6.9 ± 10.4	<0.001
CAL, mm	2.31 ± 1.75	1.20 ± 0.78	1.74 ± 0.77	<0.001	1.16 ± 0.73	1.73 ± 0.75	<0.001
Extent CAL ≥3 mm, %	40.3 ± 34.7	16.7 ± 18.9	21.1 ± 20.0	0.003	15.6 ± 18.0	20.9 ± 19.4	0.001
Gingiva above CEJ, mm	0.17 ± 1.26	0.83 ± 0.61	0.56 ± 0.61	<0.001	0.85 ± 0.59	0.58 ± 0.58	<0.001
No. of filled teeth	2.9 ± 1.9	2.5 ± 2.0	3.3 ± 2.1	<0.001	2.5 ± 2.0	3.4 ± 2.1	<0.001

Data are presented as mean ± SD or No. (%). *P* values were obtained via paired *t* tests (continuous variables), McNemar’s tests (dichotomous variables), and Wilcoxon signed rank tests (ordinal variables).

CAL, clinical attachment level; CEJ, cementoenamel junction; HbA1c, glycated hemoglobin; PD, probing depth; SHIP, Study of Health in Pomerania.

a All participants of SHIP-0 with complete information on the presented variables.

b Subgroup of dentate participants with complete periodontal examination in the maxilla.

Restricted to maxillary measurements, the number of teeth decreased by 0.2 teeth; mean PD increased from 2.03 ± 0.39 mm to 2.31 ± 0.41 mm; and mean CAL increased from 1.20 ± 0.78 mm to 1.74 ± 0.77 mm during 16 y. Mean GH decreased from 0.83 ± 0.61 mm to 0.56 ± 0.61 mm. Change in continuous eruption was 0.33 ± 0.3 mm, equaling an average yearly continuous eruption of 0.021 mm. No significant discrepancies in continuous eruption levels were found among the 3 analyzed occlusal planes (Appendix Table 3). Out of 140 participants, 4 experienced >1 mm of continuous eruption.

Next, we associated changes in point-to-plane distances with changes in periodontal variables (Table 2, Appendix Fig. 3). Based on the main models (model 1), associations of continuous eruption with periodontal outcomes were consistently nonsignificant except for GH above the CEJ (*B* = −0.34; 95% CI, −0.65 to −0.03). By extending the view to models 2 to 4 with different adjustment sets, beta coefficients were stable, in total indicating a reduction of mean GH (*B* = −0.33 to −0.30) for higher levels of continuous eruption.

**Table 2.** Regression Models Evaluating the Associations between 16-y Changes in Point-to-Plane Distances (Exposure) and Periodontal Variables (Outcomes): All Participants and after Exclusion of Those with Incident Tooth Loss.

	Model 1^a^		Model 2^b^		Model 3^c^		Model 4^d^	
Outcome	*B* (95% CI)	*P* Value	*B* (95% CI)	*P* Value	*B* (95% CI)	*P* Value	*B* (95% CI)	*P* Value
All participants (*n* = 140)								
Mean PD, mm	−0.17 (−0.41 to 0.07)	0.17	−0.20 (−0.43 to 0.04)	0.10	−0.18 (−0.42 to 0.06)	0.14	−0.19 (−0.43 to 0.04)	0.11
Extent PD ≥4 mm, %	−1.49 (−8.36 to 5.37)	0.67	−1.72 (−8.49 to 5.05)	0.62	−1.11 (−7.76 to 5.54)	0.74	−1.91 (−8.14 to 4.33)	0.55
Mean CAL, mm	0.15 (−0.20 to 0.51)	0.40	0.08 (−0.25 to 0.41)	0.64	0.10 (−0.24 to 0.44)	0.57	0.10 (−0.26 to 0.46)	0.58
Extent CAL ≥3 mm, %	5.73 (−4.26 to 15.73)	0.26	4.23 (−5.18 to 13.63)	0.38	4.49 (−5.26 to 14.25)	0.37	4.58 (−5.91 to 15.08)	0.39
Mean GH above CEJ, mm	−0.34 (−0.65 to −0.03)	0.030	−0.30 (−0.59 to −0.003)	0.048	−0.30 (−0.59 to −0.01)	0.043	−0.33 (−0.65 to −0.02)	0.036
Tooth loss (at least 1)	3.87 (0.62 to 24.13)^e^	0.15	3.57 (0.54 to 23.56)^e^	0.19	3.91 (0.52 to 29.37)^e^	0.18	4.01 (0.44 to 36.33)^e^	0.22
Excluding those with incident tooth loss (*n* = 113)								
Mean PD, mm	−0.24 (−0.51 to 0.03)	0.08	−0.30 (−0.55 to −0.04)	0.02	−0.27 (−0.53 to −0.02)	0.038	−0.33 (−0.60 to −0.06)	0.017
Extent PD ≥4 mm, %	−4.45 (−11.66 to 2.76)	0.23	−4.94 (−12.46 to 2.58)	0.20	−4.24 (−11.29 to 2.81)	0.24	−6.40 (−13.51 to 0.70)	0.08
Mean CAL, mm	0.12 (−0.33 to 0.56)	0.61	−0.06 (−0.44 to 0.33)	0.76	−0.04 (−0.44 to 0.35)	0.83	−0.04 (−0.49 to 0.42)	0.87
Extent CAL ≥3 mm, %	4.12 (−7.50 to 15.73)	0.49	0.66 (−9.87 to 11.18)	0.90	0.92 (−9.56 to 11.41)	0.86	0.31 (−11.89 to 12.52)	0.96
Mean GH above CEJ, mm	−0.37 (−0.76 to 0.01)	0.06	−0.24 (−0.59 to 0.12)	0.19	−0.24 (−0.60 to 0.11)	0.18	−0.32 (−0.72 to 0.07)	0.11

All models included the follow-up time (logarithmized; as offset variable).

CAL, clinical attachment level; CEJ, cementoenamel junction; GH, gingival height; PD, probing depth.

a Model 1 (main model): adjusted for age, sex, school education, number of filled teeth.

b Model 2: age, sex, number of filled teeth, Ekfeldt score.

c Model 3: age, sex, number of filled teeth, canine facet size.

d Model 4: age, sex, number of filled teeth, Ekfeldt score, canine facet size, school education, smoking status, abdominal obesity, diabetes mellitus, dental check-up during the last year, tooth brushing frequency and orthodontic treatment (fully adjusted model).

e Values are presented as odds ratio (95% CI).

When participants with incident tooth loss were excluded (Appendix Table 4), distances between occlusal planes and reference points remained mostly unchanged. However, associations between continuous eruption and change in mean GH turned nonsignificant for the main model, although effect estimates were consistent in direction and size (*B* = −0.37; 95% CI, −0.76 to 0.02; Table 2 lower part). Also, continuous eruption was now linked to change in mean PD in models 2 to 4 but not in the main model.

## Discussion

In this study, continuous eruption of teeth was clearly detectable. It amounted to 0.33 mm over 16 y, equaling a yearly continuous eruption of 0.021 mm. Associations of continuous eruption with periodontal outcomes were significant only for GH above the CEJ, indicating lower changes in mean GH with higher levels of continuous eruption. However, because of the relatively small effect sizes and the fact that the association was not confirmed in sensitivity analyses for which participants with tooth loss were excluded, caution is warranted when interpreting the results. Nonetheless, it should be emphasized that continuous tooth eruption occurs as a lifetime process and that the time span of 16 y covers a small part of the human adulthood. Therefore, large-scale longitudinal studies are needed to evaluate continuous tooth eruption patterns in younger and older populations.

Continuous eruption seems to be a very individual phenomenon with wide variation (Appendix Fig. 4). The total eruption rate of 0.021 mm/y is in accordance with the literature, albeit on the lower end. In particular, studies performed on front teeth and younger adults demonstrated rates up to 0.21 mm/y (Kawanami et al. 1999; Thilander et al. 1999; Bernard et al. 2004). Surveys that included posterior teeth and older participants revealed eruption rates between 0.033 and 0.1 mm/y (Whittaker et al. 1990; Craddock et al. 2007; Thilander 2009; Papageorgiou et al. 2018; Polymeri et al. 2020), which are closer to our rate. These findings support the conclusion that continuous eruption occurs with varying rates depending on individual age and possibly the presence and intensity of occlusal contacts (Christou and Kiliaridis 2007; Kiliaridis et al. 2019).

Our results give weak support to the hypothesis that, concomitant to continuous eruption, GH is reduced, thereby leading to gingival recession. This is in accordance with a 10-y follow-up study demonstrating an average tooth eruption of 0.32 mm with an average gingival recession of 0.29 mm (Huanca Ghislanzoni et al. 2017). Continuous eruption might contribute to the etiology of recessions, suggesting that increased recession is not only the result of an inflammatory process but also part of the physiologic aging process. This conclusion supports the notion that continuous eruption has to be seen in a wider perspective: a recent study on SHIP data showed that long, narrow face morphology was related to more recession (Salti et al. 2017) and a smaller masseter thickness. However, reduced bite force favors continuous eruption (Kiliaridis et al. 2019). Putting these pieces of information together, we assume that participants with long, narrow face morphologies have smaller masticatory muscles (Satiroglu et al. 2005), which in turn results in more continuous eruption. To prove this hypothesis, larger samples with more detailed variable assessments are necessary.

The sensitivity analysis excluding participants with incident tooth loss, however, showed a minimal decrease of 0.02 to 0.03 mm of vertical dimension measurements (Appendix Table 4). Thus, we ruled out that vertical measurements (exposure) were biased due to tipping or elongation of teeth in SHIP-3. Furthermore, extraction affects the periodontal situation on adjacent teeth (Grassi et al. 1987). However, on average, the periodontal situation in the total sample was comparable to that of the subsample. Also, effect estimates were broadly consistent with main analyses.

Some strengths and limitations in this study merit consideration. One strength is that by averaging continuous eruption of multiple teeth, the measurements were unsusceptible to errors from single outliers (e.g., single tooth tipping or elongation). Thus, we could prove that all teeth were affected by continuous eruption, whereas other studies were often limited to incisors. However, we could not make any statements for specific teeth because analyses were conducted on a participant level. Importantly, this was the first prospective cohort study with a reasonable sample size to evaluate associations between continuous eruption and periodontal outcome, including quality controls and data monitoring to ensure high-quality measurements and data recordings. Nevertheless, our sample is still limited and might have been too small and preselected to detect more distinct correlations. Additionally, periodontal measurements were restricted to those taken half-mouth in the maxilla at 4 sites. Thus, periodontal severity estimates were probably underestimated, and effect sizes were shifted toward the null (Akinkugbe et al. 2015). Thus, “true” effect sizes might have been larger. Furthermore, clinical methods assessing the periodontal status were probably too insensitive. While PD and CAL were clinically quantified via a manual periodontal probe with rounding to the nearest millimeter, continuous eruption was quantified virtually by vector analysis measuring values more precise than 0.01 mm. Thus, the “noise” of the periodontal measurements was potentially too large, and the estimated 16-y changes were probably too imprecise. Future studies should measure continuous eruption with a higher resolution and analyze correlations on the tooth level to disentangle physiologic versus inflammatory reactions of the gingiva (Kiliaridis et al. 2019).

Continuous eruption had been observed for many years but was subject to repeated controversial discussion. Over 16 y, continuous tooth eruption averaged to 0.33 mm, corresponding to 0.021 mm/y. This study provides minor evidence that continuous eruption might lead to “physiologic” recession. Increases in continuous eruption, although small on average, might have a significant impact on functional, aesthetic, and periodontal parameters in the long term, although it does not affect all patients to the same degree. To detect more detailed interdependencies and to identify participants at higher risk for continuous eruption, large-scale cohort studies are required.

## Author Contributions

C. Wiedemann, contributed to data interpretation, drafted the manuscript; C. Pink, S. Samietz, E. Schulz-Kornas, contributed to data analysis and interpretation, critically revised the manuscript; A. Daboul, contributed to design and data interpretation, critically revised the manuscript; H. Völzke, contributed to data acquisition, critically revised the manuscript; K.F. Krey, contributed to design, critically revised the manuscript; B. Holtfreter, contributed to data analysis and interpretation, drafted the manuscript; T. Kocher, contributed to conception, design, and data acquisition, drafted the manuscript. All authors gave final approval and agree to be accountable for all aspects of the work.

## Supplemental Material

sj-pdf-1-jdr-10.1177_0022034521999363 – Supplemental material for Is Continuous Eruption Related to Periodontal Changes? A 16-Year Follow-up Click here for additional data file. Supplemental material, sj-pdf-1-jdr-10.1177_0022034521999363 for Is Continuous Eruption Related to Periodontal Changes? A 16-Year Follow-up by C. Wiedemann, C. Pink, A. Daboul, S. Samietz, H. Völzke, E. Schulz-Kornas, K.F. Krey, B. Holtfreter and T. Kocher in Journal of Dental Research
