# Supplementary material for: Is Continuous Eruption Related to Periodontal Changes? A 16-Year Follow-up
Source: J Dent Res. 2021 Mar 3;100(8):875–82. doi: 10.1177/0022034521999363 (PMC8258728; doi:10.1177/0022034521999363)
Supplement: sj-pdf-1-jdr-10.1177_0022034521999363 – Supplemental material for Is Continuous Eruption Related to Periodontal Changes? A 16-Year Follow-up [file sj-pdf-1-jdr-10.1177_0022034521999363.pdf]

Online Supplemental Appendix

Is continuous eruption related to periodontal changes? A 16-year follow up

Conrad Wiedemann, Christiane Pink, Amro Daboul, Stefanie Samietz, Henry Völzke,

Ellen Schulz-Kornas, Karl-Friedrich Krey, Birte Holtfreter, Thomas Kocher

Material and methods

Covariates

Socio-demographic variables were obtained through health-related interviews. School education was categorized as <10/10/>10 years. Smoking was classified into never, former, and current smoking. Information on dental check-up during the last 12 months, tooth brushing frequency (</≥2 times/day), and former orthodontic treatment were recorded. Anthropometric measurements were taken under standardized conditions using a weighing scale and a height measuring devices (SOEHNLE, Murrhardt, Germany). Waist circumference was measured to the nearest 0.5 cm. Abdominal obesity was defined as a waist circumference of ≥102 cm for men and ≥88 cm for women. Diabetes mellitus was defined as self-reported physician's diagnosis or antidiabetic treatment (Anatomical Therapeutic Chemical Classification System (ATC) code A10) or non-fasting glucose levels ≥11.1 mmol/l or glycated haemoglobin concentrations ≥6.5%.

Coronal caries examinations

Recording protocols were identical in SHIP-0 and SHIP-3, with the same side being examined. All examinations were conducted in an illuminated dental chair and without the use of aspiration or an air jet. Coronal caries was diagnosed visually using a periodontal probe (PCP-11, Hu-Friedy, USA) to touch the tooth surface softly. Coronal caries was examined excluding third molars on a surface level

in order to determine the number of sound, carious (including dentine caries only), missing and filled surfaces in a half-mouth design (alternating on the left or right side) after no statistically relevant right-left difference was detected in the pilot phase of SHIP-0. The number of filled teeth was determined based on with a maximum of 7 permanent teeth (upper jaw only, excluding third molars) with (depending on the tooth type) 4 to 5 surfaces each, resulting in 32 surfaces being assessed in total.

In SHIP-0, dental examinations were performed by eight calibrated and licensed general dentists. In calibration exercises, Cohen's kappa reliability coefficients of 0.9-1.0 (intra-examiner) and 0.93-0.96 (inter-examiner) were attained. In SHIP-3, dental examinations were performed by six calibrated and licensed general dentists. In calibration exercises, Cohen's kappa reliability coefficients of 0.88-0.98 (intra-examiner) and 0.95-0.99 (inter-examiner) were attained.

Tables and figures

Appendix Table 1. The predefined first-choice-reference-points.

Point	Location	Description
S000	anterior teeth	most labial point
S001	Tooth 14	medial in the central fissure
S002	Tooth 24	medial in the central fissure
S003	Tooth 11	on the palatinal fossa
S004	Tooth 12	on the palatinal fossa
S005	Tooth 22	on the palatinal fossa
S006	Tooth 13	on the palatinal fossa
S007	Tooth 23	on the palatinal fossa
S008	Tooth 15	medial in the central fissure
S009	Tooth 25	medial in the central fissure
S010	Tooth 16	intersection of central fissure and buccal fissure
S011	Tooth 26	intersection of central fissure and buccal fissure
S012	Tooth 17	intersection of central fissure and buccal fissure
S013	Tooth 27	intersection of central fissure and buccal fissure
S014	median palatine raphe	distal end
S015	most distal palatine ruga in the I. quadrant	medial end
S016	most distal palatine ruga in the II. quadrant	medial end

Appendix Table 2. Points of different choices defining the occlusal planes.

Occlusal plane	Priority	Point 1	Point 2	Point 3
1	1. choice	16	12	24
	2. choice	17	13	25
	3. choice	15	11	26
2	1. choice	14	22	26
	2. choice	15	23	27
	3. choice	16	11	25
3	1. choice	17	13	25
	2. choice	15	23	27
	3. choice	16	11	26

Appendix Table 3. Distances between occlusal planes and reference points in SHIP-0 and SHIP-3.

Occlusal plane	Reference Point	N	Distance in SHIP-0, mm	Distance in SHIP-3, mm	16-year change, mm	P-value
1	14	133	13.32 ± 2.83	13.66 ± 2.86	0.33 [0.28; 0.39]	<0.001
1	15	140	12.07 ± 2.59	12.41 ± 2.58	0.34 [0.28; 0.40]	<0.001
1	16	140	12.22 ± 2.42	12.54 ± 2.46	0.33 [0.27; 0.39]	<0.001
1	All	140	12.49 ± 2.39	12.83 ± 2.41	0.33 [0.28; 0.39]	<0.001
2	14	133	13.44 ± 2.78	13.78 ± 2.81	0.34 [0.28; 0.39]	<0.001
2	15	140	12.25 ± 2.53	12.59 ± 2.54	0.34 [0.28; 0.39]	<0.001
2	16	140	12.20 ± 2.33	12.54 ± 2.37	0.33 [0.28; 0.39]	<0.001
2	All	140	12.58 ± 2.34	12.92 ± 2.37	0.33 [0.28; 0.39]	<0.001
3	14	133	13.24 ± 2.70	13.57 ± 2.72	0.33 [0.27; 0.39]	<0.001
3	15	140	12.03 ± 2.54	12.36 ± 2.53	0.34 [0.28; 0.39]	<0.001
3	16	140	12.19 ± 2.41	12.50 ± 2.43	0.32 [0.27; 0.38]	<0.001
3	All	140	12.44 ± 2.36	12.77 ± 2.37	0.33 [0.28; 0.38]	<0.001
all	All	140	12.51 ± 2.31	12.84 ± 2.33	0.33 [0.28; 0.38]	<0.001

Values of point-to-plane distances are presented as mean ± standard deviation. Changes in point-to-plane distances between SHIP-0 and SHIP-3 are presented as mean [95% confidence interval]. P-values were obtained by paired t-tests.

Appendix Table 4. Distances between occlusal planes and reference points in SHIP-0 and SHIP-3, excluding participants with incident tooth loss.

Occlusal plane	Reference point	N	Distance in SHIP-0, mm	Distance in SHIP-3, mm	16-year change, mm	P-value
1	14	106	13.35 ± 2.95	13.65 ± 2.97	0.30 [0.24; 0.36]	<0.001
1	15	113	11.99 ± 2.67	12.30 ± 2.67	0.31 [0.25; 0.37]	<0.001
1	16	113	12.25 ± 2.45	12.54 ± 2.48	0.29 [0.24; 0.35]	<0.001
1	all	113	12.48 ± 2.46	12.78 ± 2.48	0.30 [0.25; 0.35]	<0.001
2	14	106	13.49 ± 2.89	13.79 ± 2.92	0.30 [0.24; 0.36]	<0.001
2	15	113	12.18 ± 2.61	12.48 ± 2.62	0.30 [0.24; 0.36]	<0.001
2	16	113	12.24 ± 2.37	12.54 ± 2.41	0.30 [0.24; 0.36]	<0.001
2	all	113	12.57 ± 2.41	12.87 ± 2.44	0.30 [0.25; 0.36]	<0.001
3	14	106	13.26 ± 2.77	13.57 ± 2.80	0.31 [0.25; 0.38]	<0.001
3	15	113	11.93 ± 2.60	12.26 ± 2.59	0.32 [0.26; 0.39]	<0.001
3	16	113	12.22 ± 2.39	12.52 ± 2.42	0.30 [0.24; 0.37]	<0.001
3	all	113	12.41 ± 2.39	12.72 ± 2.41	0.31 [0.25; 0.37]	<0.001
all	all	113	12.49 ± 2.37	12.79 ± 2.39	0.31 [0.25; 0.36]	<0.001

Values of point-to-plane distances are presented as mean ± standard deviation. Changes in point-to-plane distances between SHIP-0 and SHIP-3 are presented as mean [95% confidence interval]. P-values were obtained by paired t-tests.

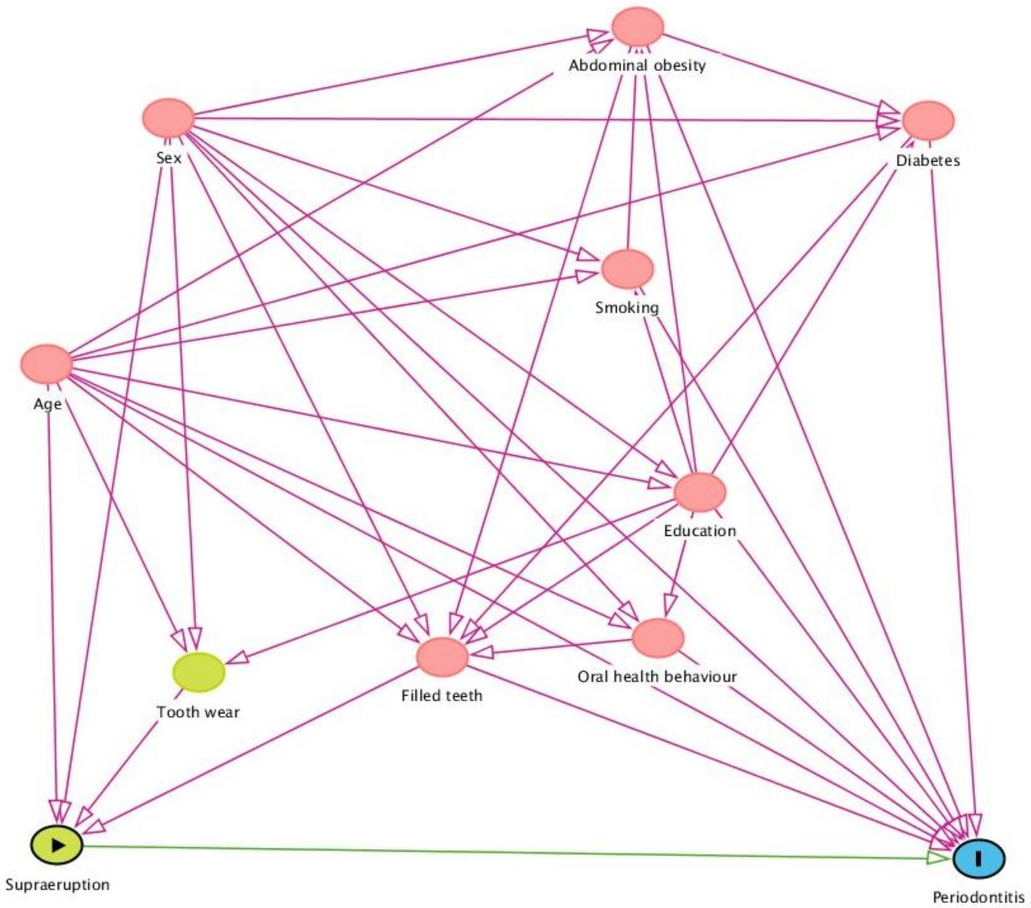

Appendix Figure 1. Directed Acyclic Graph excluding the association between tooth wear and periodontitis. Minimal sufficient adjustment sets: {age, sex, education, number of filled teeth} or {age, sex, number of filled teeth, tooth wear}.

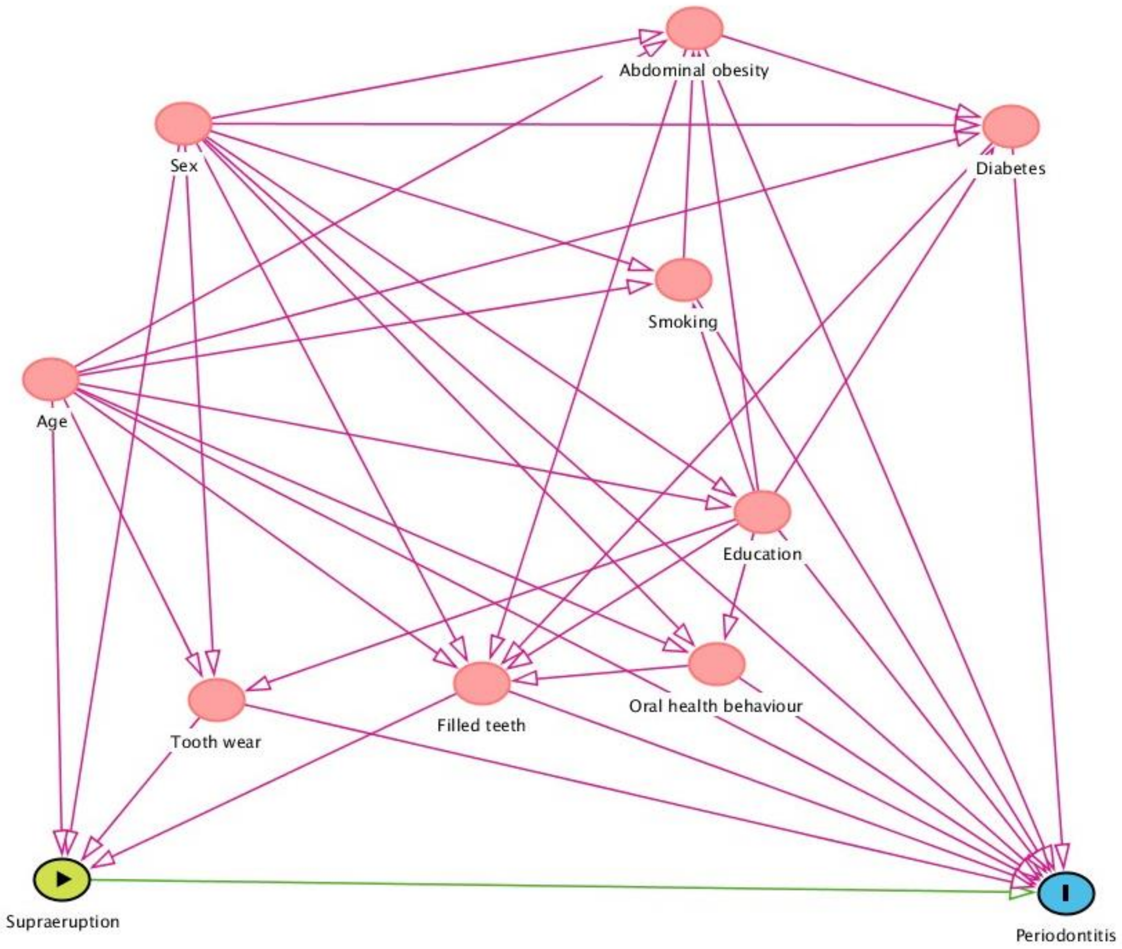

Appendix Figure 2. Directed Acyclic Graph, including the association between tooth wear and periodontitis. Minimal sufficient adjustment set: {age, sex, number of filled teeth, tooth wear}.

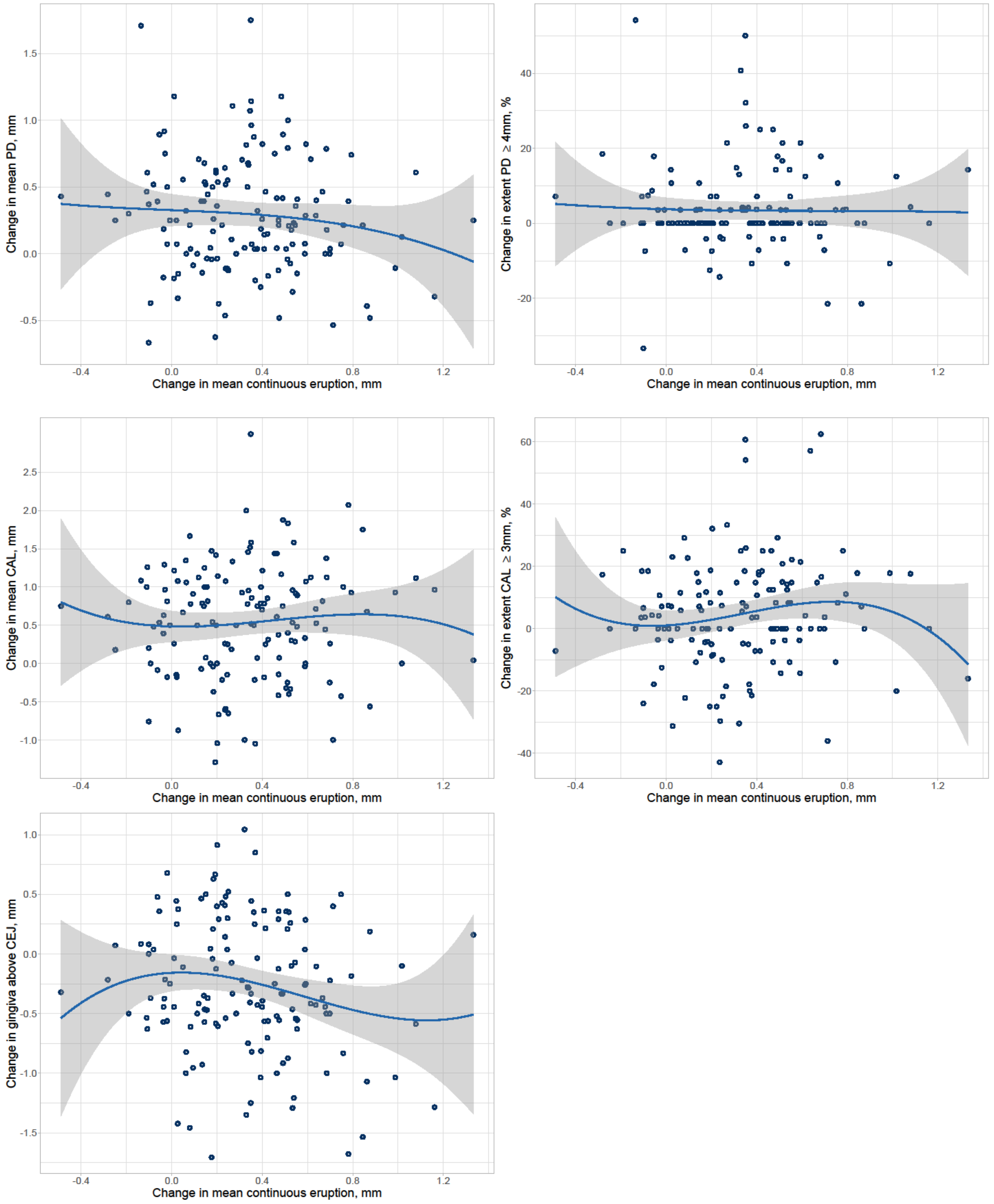

Appendix Figure 3. Scatter-plots of change in mean probing depth (PD), extent PD ≥4 mm, mean clinical attachment levels (CAL), extent CAL ≥3 mm, and mean gingiva above cemento-enamel junction (CEJ) according to change in mean continuous eruption. A lowess smoother (blue line) and the corresponding 95% confidence bands were added (grey shading).

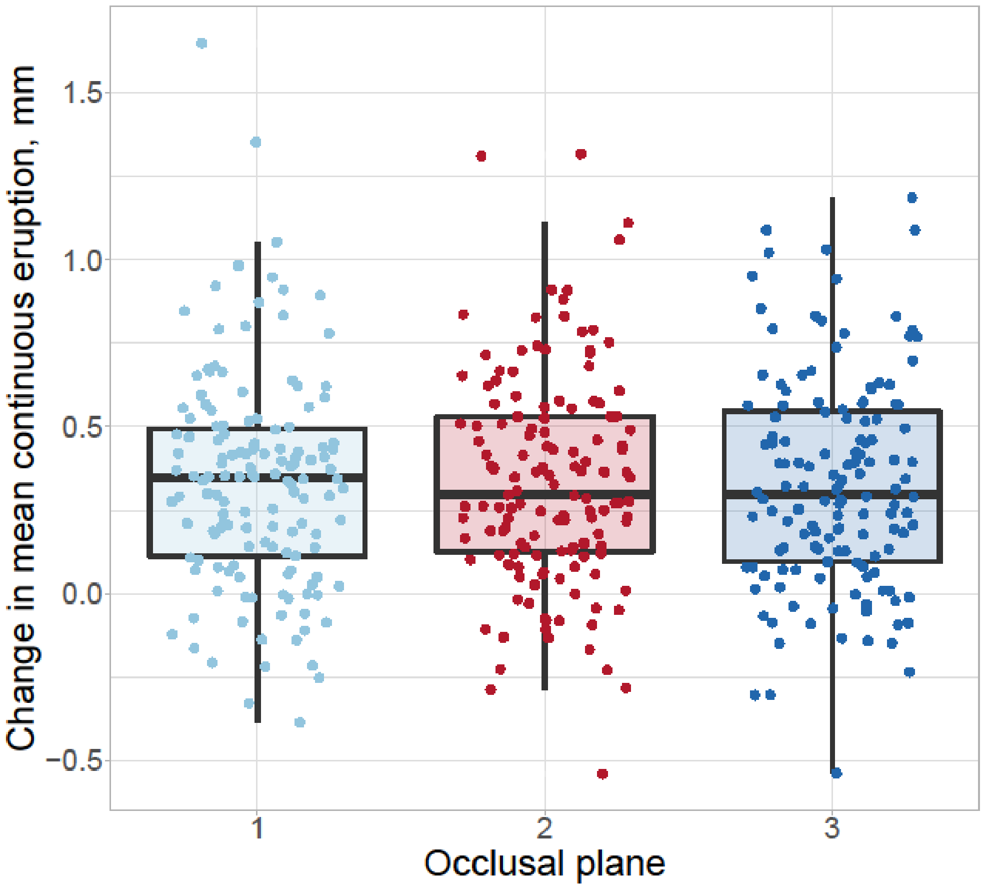

Appendix Figure 4. Box-plots of 16-year changes in continuous eruption according to occlusal planes.
